# The complete mitochondrial genome of *Pycanum ochraceum* Distant 1893 (Hemiptera: Tessaratomidae)

**DOI:** 10.1080/23802359.2021.1997659

**Published:** 2021-11-10

**Authors:** Yu-Chen Wang, Gen-Li Li, Xin-Yang Liu, Qiu-Ju He, Chuan-Hui Yi, Chen Yang, En-Jiao Zhu

**Affiliations:** a Key Lab Forest Disaster Warning and Control Yunnan, Southwest Forestry University, Kunming, China; b Yunnan Academy of Biodiversity, Southwest Forestry University, Kunming, China; c Yunnan Forestry Technological College, Kunming, China

**Keywords:** *Pycanum ochraceum*, Tessaratomidae, mitochondrial genome, phylogenetic analysis

## Abstract

In the present study, the complete mitochondrial genome of *Pycanum ochraceum* was identified for the first time. The entire genome is 17,198 bp in length with 73.6% A + T content. It contains 22 transfer RNA genes (tRNAs), 2 ribosomal RNA genes (rRNAs), 13 protein-coding genes (PCGs) and 1 noncoding control region (D-loop). Phylogenetic analysis showed that Tessaratomidae bugs are monophyletic. This study can provide essential DNA molecular data for further phylogenetic and evolutionary analysis for Heteroptera.

*Pycanum ochraceum* Distant, 1893 is one of the most economically important pests in agriculture and gardening, which mainly damages Gramineae, Leguminosae, and Malvaceae plants in southwestern China and neighboring countries (Li et al. 2005). So far, there is only one mitochondrial gene (*COX1*) of *P. ochraceum* has been reported (Li et al. 2005). In the present study, we identified the complete mitogenome of *P. ochraceum* for the first time, which will enrich the mitochondrial genome database of Tessaratomidae, and provide a molecular basis for the future research of this pest.

In this study, *P. ochraceum* was collected in Xiangcaihe, Zhonghe Town, Tengchong City, Yunnan Province, China (25°04′06″N, 98°24′20″E) on 20 September 2020. The specimen (voucher code: PO_002_2020) was deposited in the in the herbarium of Southwest Forestry University, Kunming, China (Collector: Chuan-Hui Yi, ynkcx2007@163.com, identified by: Chuan-Hui Yi). Genomic DNA was extracted from the thorax muscle of a single adult using the Sangon Animal genome DNA Extraction Kit (Shanghai, China). The isolated DNA was sheared to 500-bp fragments in a Covaris (KBiosciences) ultrasonicator device for preparing the next-generation sequencing (NGS) library using the paired-end NEBNext Ultra DNA Library Prep Kit for Illumina (Illumina, San Diego, CA). The library was sequenced using NovaSeq (Illumina, San Diego, CA). Clean reads were de novo assembly by using commercial software (Geneious V8, Auckland, New Zealand) to produce a single, circular form of complete mitogenome. The rRNA, tRNA and protein coding genes of *P. ochraceum* mitogenome were predicted by using MITOS (Bernt et al. 2013), DOGMA (Wyman et al. 2004) and ARWEN (Laslett and Canbäck 2008) software.

The complete mitochondrial genome of *P. ochraceum* is a circular genome with 17,198 bp in length (Genbank: MW899159), which contains 13 protein-coding genes (PCGs), 22 transfer RNAs (tRNAs), 2 ribosomal RNA genes, and 1 noncoding control region. The overall nucleotide composition showed a strong AT bias, with 73.6% of A + T content (A 41.9%, T 31.7%, C 15.0%, and G 11.4%). Four PCGs (*ND1*, *ND4*, *ND5*, and *ND4L*) were encoded by the J-strand (majority strand), and nine PCGs (*ND2*, *ND3*, *ND6*, *COX1*, *COX2*, *COX3*, *CYTB*, *ATP6*, and *ATP8*) were encoded by the N-strand (minority strand). The lowest A + T content is in *COX1* with 68.1%, while the highest is in *ND4L* with 78.0%. Eleven PCGs start with a typical ATN codon, including four with ATA (*ND2*, *COX2*, *ND3* and *ND5*), six with ATG (*ATP6*, *ATP8*, *COX3*, *NAD4*, *NAD6* and *CYTB*) and one start with TTG (*COX1*); while *ND4L* start with ATT and *ND1* start with GTG. Most PCGs of *P. ochraceum* have the conventional complete stop codons T-, including seven with TAA (*ATP8*, *ND3*, *ND5*, *ND4*, *ND4L*, *ND6*, and *ND1*) and three with TAG (*ND2*, *ATP6*, and *CYTB*); while three PCGs end with the incomplete stop codon T- (*COX1*, *COX2*, and *COX3*).

Phylogenetic analysis was performed based on the nucleotide sequences of complete mitochondrial genome of 43 species in 11 families from Heteroptera, and two species in one family (outgroup) from Homoptera (Jiang 2017). Phylogenetic tree was constructed by IQ-TREE (Nguyen et al. 2015) in PhyloSuite v1.2.2 (Zhang et al. 2020) based on concatenated 13 protein-coding genes (PCGs) by using the Maximum Likelihood (ML) method with 10,000 ultrafast bootstrap replicates. The results showed that *P. ochraceum* has the closest evolutionary relationship with *Tessaratoma papillosa* and *Mattiphus splendidus* in Tessaratomidae, and the clade of Tessaratomidae were correctly identified in Heteroptera with high bootstrap values (Figure 1). The complete mitochondrial genome data of *P. ochraceum* present in this study will provide useful genetic data for further phylogenetic and evolutionary analysis for Heteroptera.

**Figure 1.**
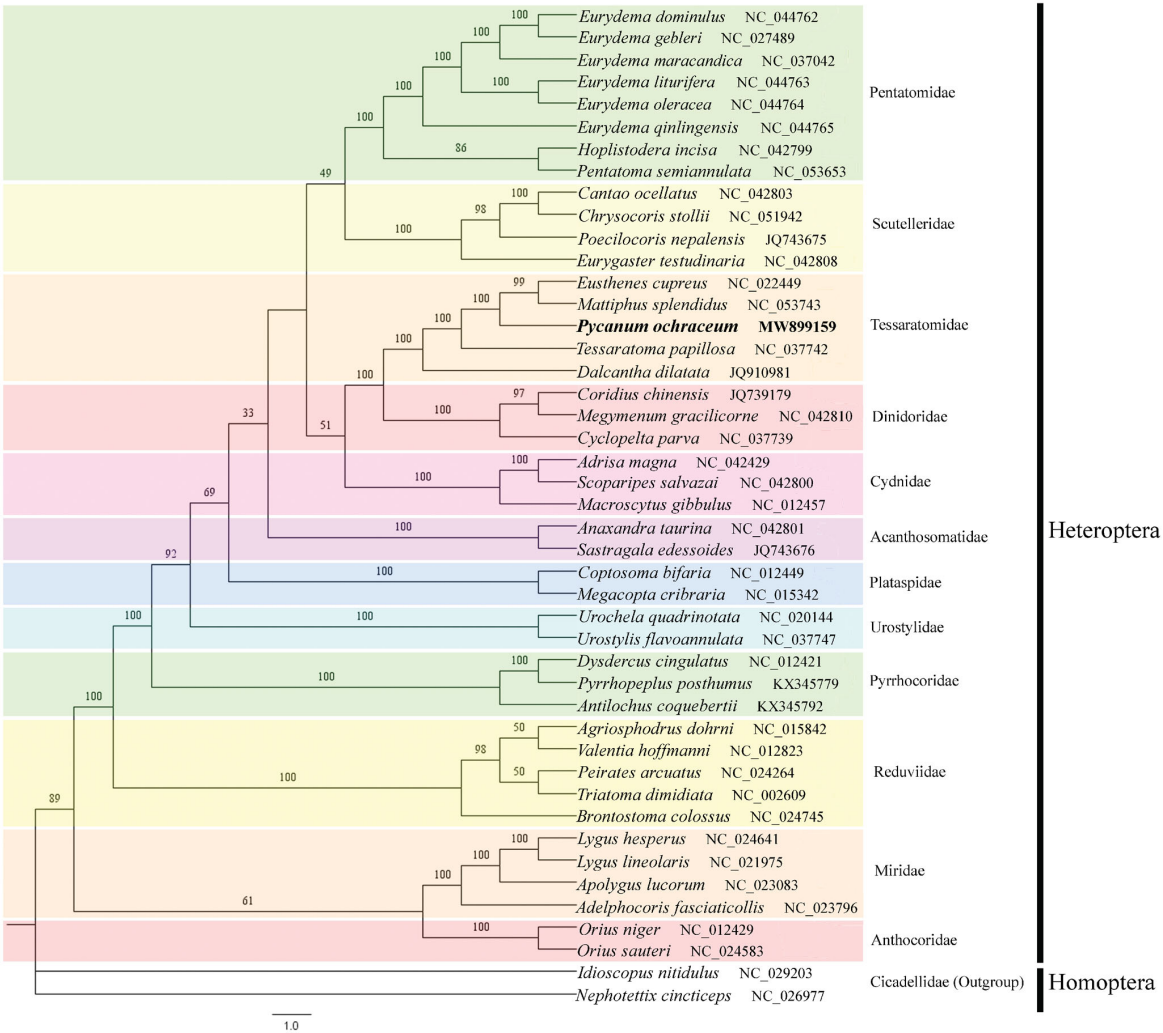
Phylogenetic tree for *P. ochraceum* and the related species. Numbers above branches represent Bootstrap support values. The position of *P. ochraceum* is marked with bold fonts.

## Data Availability

Mitogenome data supporting this study are openly available in GenBank at: https://www.ncbi.nlm.nih.gov/ MW899159. The associated BioProject, Sequence Read Archive (SRA), and Bio-sample accession numbers are PRJNA754508, SRR15461509, and SAMN20792569, respectively.
